# The 2011 Ming K Jeang Award for Excellence in *Cell & Bioscience*

**DOI:** 10.1186/2045-3701-2-16

**Published:** 2012-05-08

**Authors:** Yun-Bo Shi

**Affiliations:** 1 The National Institutes of Health, Bethesda, MD, USA

## Abstract

Two research groups led by Dr T.C. Wu of Johns Hopkins Medical Institutions and Dr P. Liu of National Human Genome Research Institute, National Institutes of Health, respectively, have won the 2011 Ming K Jeang Award for Excellence in *Cell & Bioscience.*

## Editorial

We are very pleased to announce that two research groups, who each published an outstanding research article in *Cell & Bioscience* in 2011, have been selected to receive the Ming K Jeang Award for Excellence in *Cell & Bioscience*. The Ming K Jeang Award for Excellence in *Cell & Bioscience* was established in 2011 with a generous donation from the Ming K Jeang Foundation to honor outstanding research articles published in *Cell & Bioscience*, the official journal of the Society of Chinese Bioscientists in America (SCBA; http://www.scbasociety.org). A committee of *Cell & Bioscience* Editors, chaired by Dr Chris Lau, considered all research articles published in the journal in 2011 and produced a short list of 4 articles. The committee then voted the following two articles to receive the award [1,2]:

DNA vaccines delivered by human papillomavirus pseudovirions as a promising approach for generating antigen-specific CD8+ T cell immunity

Shiwen Peng, Barbara Ma, Shu-Hsia Chen, Chien-Fu Hung, TC Wu

*Cell & Bioscience* 2011, 1:26 (28 July 2011)

Transient knockdown and overexpression reveal a developmental role for the zebrafish enosf1b gene

Steve Finckbeiner, Pin-Joe Ko, Blake Carrington, Raman Sood, Kenneth Gross, Bruce Dolnick, Janice Sufrin, Paul Liu

*Cell & Bioscience* 2011, 1:32 (26 September 2011)

Congratulations to these two groups of investigators for jobs well done! We are also looking forward to receiving contributions of outstanding research articles from the scientific community in 2012 and beyond.
